# What Can Mercury Teach Us About Membranous Nephropathy and Minimal Change Disease?

**DOI:** 10.1016/j.ekir.2022.04.078

**Published:** 2022-04-22

**Authors:** Tiffany N. Caza, Laith F. Al-Rabadi

**Affiliations:** 1 Arkana Laboratories, Little Rock, Arkansas, USA; 2 Division of Nephrology, Department of Medicine, University of Utah, Salt Lake City, Utah, USA

See Clinical Research on Page 1189

From clinical associations of environmental exposure beginning with the story of cationic bovine serum in pediatric membranous nephropathy (MN) more than a decade ago^1^ and recently to the association of lipoic acid with NELL1-associated MN,^2^ the impact of environment in triggering autoimmunity warrants a fresh examination given the increased influence of anthropogenic factors on the development of glomerular disease. Repeated exposure to heavy metal compounds through pollution, cosmetics, vaccines, and manufacturing processes is associated with nephrotoxicity, which can manifest as minimal change disease (MCD) or MN. Environmental toxins are likely under-recognized triggers of glomerular disease. Exposure to mercury (Hg) and other environmental toxins is often not elucidated in a patient’s social history, as most of the exposures do not arise from known occupational hazards. This is further compounded by the rarity of Hg poisoning overall. Recognition is critical, as patients may have spontaneous remission and a good prognosis with exposure cessation and chelation therapy.^3^ Conversely, sustained chronic Hg poisoning results in kidney damage from sustained proteinuria with a lack of response to immunosuppression.

Recent work from Gao *et al.*^3^ investigated the largest cohort to date of glomerular disease due to Hg poisoning. They demonstrated from a group of 172 patients that renal toxicity occurs in more than one-quarter of patients with chronic Hg intoxication. Patients with higher serum and urinary Hg concentrations developed glomerular disease compared with patients without renal involvement, suggesting development of nephrotic syndrome is more common in patients with higher levels or duration of exposure.^3^ The authors sought to increase awareness of this important trigger of MCD and MN and subsequently elucidated explanatory sources of exposure, demographics, and outcomes.

Prior studies revealed that chronic Hg poisoning arises from multiple potential sources and routes of exposure, although limited to case reports and small series. Exposures can be due to different forms of Hg, including organic (phenylmercury or methylmercury), inorganic (Hg^1+^ mercurous or Hg^2+^ mercuric salts), and elemental species. Modes of entry include inhalation, absorption through the skin or mucous membranes, or ingestion. Sources of exposure include inhalation during fluorescent lightbulb recycling,^4^ ingestion of herbal and traditional medicinal supplements,^5^^,^^6^ ingestion of freshwater fish containing methylmercury,^7^ topical exposure by hair dying agents,^6^^,^^8^ and transdermal exposure through use of facial whitening creams,^6^^,^^8^^,^^9^^,^S1–S4 of which was the most common exposure type within the study of Gao *et al.*^3^ (Figure 1). Traditional medicines from Ayurveda, Siddha, Tibetan, Unani, and Chinese supplements can contain Hg and are the most common source of toxicity in some populations.S5 Indigenous medications associated with MN in India include Swasa Kalpa syrup and Mezhugu, which are used to treat respiratory conditions, such as allergies, asthma, and upper respiratory tract infections.^5^^,^S5 Recognition of inciting agents is required for prevention and stopping manufacture of substances with high levels of Hg. For example, dental amalgams,S6 Hg thermometers, teething powders for infants,S7 psoriasis creams,S8 and diuretics containing HgS9 were removed from the market for such reasons.

**Figure 1:**
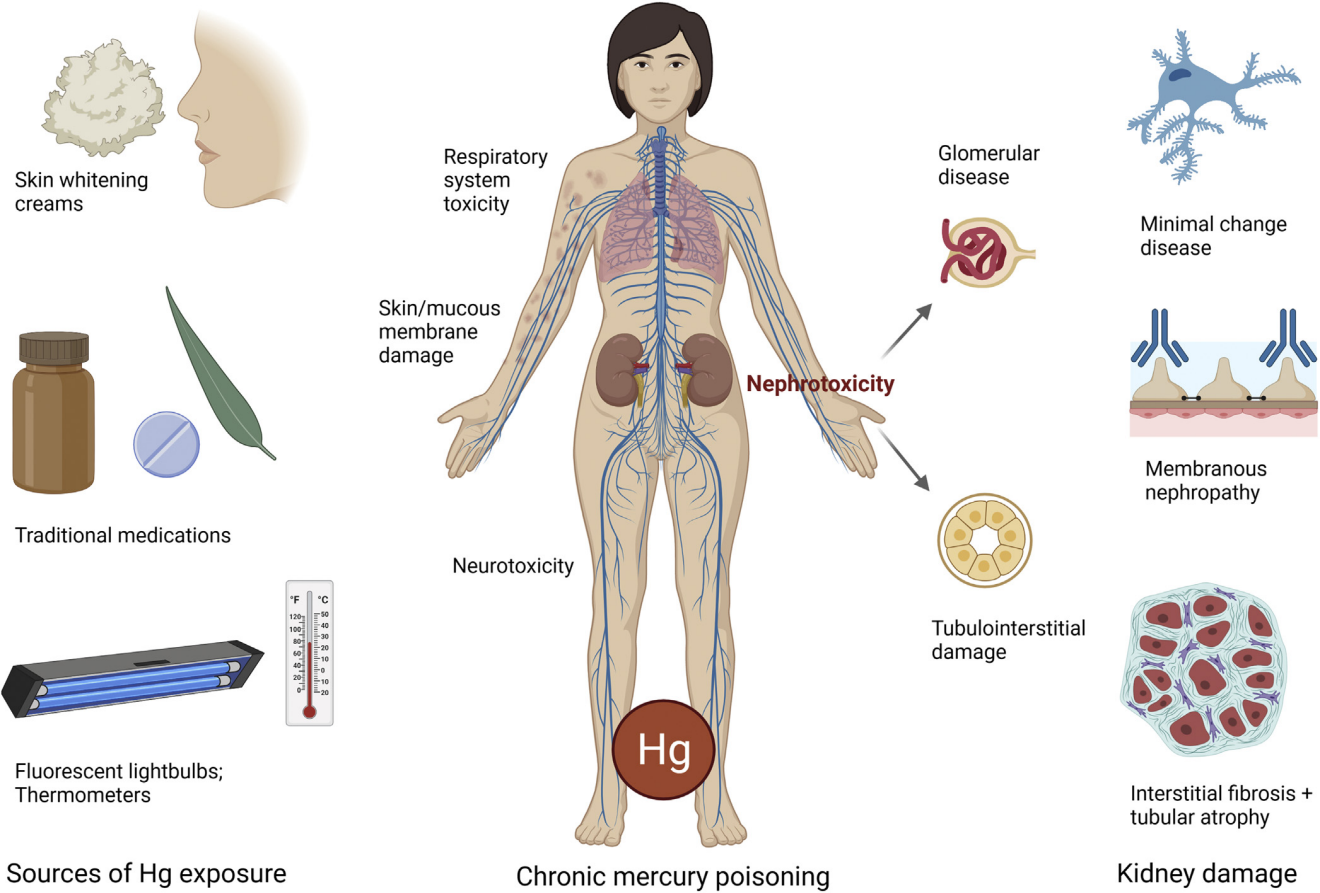
Sources and consequences of Hg exposure. Hg exposure can occur from various environmental sources, including skin whitening creams, traditional/indigenous medications, manufacture and recycling of fluorescent lightbulbs and thermometers, among others. Chronic Hg exposure over time results in Hg poisoning, of which affects multiple organ systems, including the urinary system, nervous system, respiratory system, and skin. Nephrotoxicity manifests as proteinuric glomerular disease, with development of minimal change disease or membranous nephropathy. If cessation of exposure does not occur, glomerular and tubulointerstitial scarring develops, resulting in chronic renal failure. Hg, mercury.

Chronic Hg exposure can be difficult to recognize in clinical practice due to nonspecific symptoms of toxicity, of which also may go unnoticed owing to an insidious onset of prolonged exposure. Toxicity affects sites of entry, including the central nervous system, gastrointestinal tract, skin, and mucosa, including the kidneys involved in Hg excretion (Figure 1). The most common symptoms involve the nervous system (>50% of patients), which manifests as visual disturbances, headaches, tremors, numbness, dizziness, and memory problems.S10 Psychiatric disturbances are also common, with patients experiencing mood swings, irritability, and insomnia. Dermatitis can occur at sites of transdermal exposure, and patients with exposure by inhalation of Hg vapor may develop shortness of breath. Approximately one-quarter of patients will demonstrate nephrotoxicity, of which may or may present with other symptoms of Hg intoxication.^3^^,^S10

Diagnosis of Hg-associated MCD or MN is primarily based on clinical suspicion and is often supported by measurements of serum and urine Hg levels. Hg deposits within the tissues and the levels do not necessarily reflect the actual degree of chronic Hg poisoning. For most patients with these glomerular diseases, the pretest probability of Hg toxicity would be very low. Inquiring about the above sources of exposures in the patient's history and recognition of concurrent neurologic symptoms can help identify these patients. When asking about medications, be sure to include over-the-counter drugs, supplements, and traditional/indigenous medications. Histopathologic features on biopsy can also provide clues to the inciting cause. All cases of MN due to Hg intoxication have been reported to be negative for the phospholipase A2 receptor and often reveal mesangial deposits and IgG1-dominant immune deposits (compared with IgG4 predominance found in “primary” MN).^7^ In MCD, there was reduced podocyte foot process effacement with shorter foot processes identified ultrastructurally in patients with Hg-associated disease.^6^

Populations highly exposed to Hg have a higher prevalence of autoimmune disease,S11 including kidney disease. The authors hypothesized that higher concentrations of Hg over a short duration injure the podocytes directly and result in MCD. Conversely, chronic exposures to lower levels of Hg induce MN by dysregulation of the immune system rather than direct effects.^3^^,^^8^ The kidney is first exposed to Hg through uptake at proximal tubular epithelial cellsS12 and can induce chronic tubulointerstitial damage with prolonged exposure. Hg is exceptionally known to affect different immune pathways. Potential pathophysiological mechanisms underlying Hg-induced glomerular disease have been investigated in animal models. Rodents exposed to Hg-containing compounds develop proteinuria. This occurs in some genetic backgrounds and not others, suggesting a role for genetic susceptibility. T-cells from Hg-exposed mice demonstrate a T helper 2-predominant immune response with increased production of interleukin-2 and interleukin-4.S13 Increased interleukin-4 levels promote T cell-induced B cell proliferation with production of IgG1 subclass autoantibodies in mice exposed to HgS14, the same IgG subclass of which is predominant in patients with MN due to Hg toxicity.^8^ The molecular mechanism may include increased oxidative stress by interaction with and depletion of glutathioneS15 and disrupted apoptosis,S16 which impairs T cell selection and peripheral tolerance to permit survival of autoreactive lymphocytes. Hg itself is not identified within immune complexes, suggesting immune deposits arise secondary to immune system activation.

Hg exposure results in formation of *in situ* immune complexes planted at the subepithelial space in genetically susceptible mice. Antibodies eluted from glomeruli are IgG1 and IgG2 and react with laminin within the glomerular basement membrane (GBM).S17 These antibodies were found to be pathogenic, as passive transfer of antibodies eluted from kidneys of mice with exposure to mercuric chloride into mice without exposure resulted in MN.S17 In lupus-prone mice, Hg triggers a robust autoimmune response with production of an array of autoantibodies, against double stranded DNA, fibrillarin, cardiolipin, type IV collagen, heparan sulfate proteoglycan, entactin, and phosphatidylethanolamine, among others.S18^,^S19

Antibodies reacting with basement membrane proteins in the setting of Hg exposure reveal the importance of podocyte basement membrane integrity in the development of nephrotic syndrome. Hg has high affinity binding to sulfanyl groups of proteins,S20 permitting interference with multiple cellular processes, including podocyte to GBM crosstalk, which may disturb the integrity of basement membrane through altering the protein conformation through high reactivity to thiol groups. These include proteins such as laminin and fibrillarin, glycoproteins that are present in the extracellular matrix and crucial for anchoring the cells to the basement membrane, identified in animal models induced by Hg. These targeted antigens are cationic, developing a strong interaction with negatively charged GBM components. Interestingly, other exogenous substances that induce proteinuria, including other heavy metals (gold salts, silver, cadmium, lead, and platinum),S21^,^S22 medications (such as nonsteroidal anti-inflammatory drugs, penicillamine, bucillamine, and lipoic acid),^2^^,^S23–S25 and dietary substances (including cationic bovine serum albumin)^1^ are also cationic in nature. Endogenous antigenic proteins in MN are also cationic with affinity for negatively charged GBM components.

It is possible that similar pathogenesis mechanisms may be implicated in multiple drugs and heavy metals associated with MN. Cationic substances within immune complexes could interact with negatively charged GBM components and transverse to the subepithelial space or act as weak reducing agents to modify protein folding.S26 This may incite complement, supported by an increased frequency of C4 and C1q deposition and IgG1-predominant antibodies^8^^,^S14 within immune complexes in Hg-induced MN. In addition to IgG1 predominance and C1q expression, Hg-induced MN often has mesangial immune deposits and is phospholipase A2 receptor negative, distinguishing from idiopathic MN.^7^

So, what can Hg teach the research and medical communities about MN and MCD? MN typically is presumed to result from a disruption of the immune system in genetically predisposed patients. Several reports implicate environmental factors as precipitating agents, although understanding of how such agents mediate pathogenesis requires further clarification. In patients with phospholipase A2 receptor-negative MN or in adult patients with MCD, it is critical to “dive deep” to identify an inciting cause. Environmental toxins such as Hg, medications such as nonsteroidal anti-inflammatory drugs, and infections can be reversible causes of MN and MCD, which may remit with cessation of exposure and/or chelation therapy. Without identification of the inciting trigger, further renal damage ensues, and patients may be treated with unnecessary immunosuppression, often without response. MN or MCD due to Hg intoxication has a good prognosis, but only if properly recognized and treated with chelation therapy, as in its absence, high Hg levels will persist for months.S27 Gao *et al.*^3^ shed light on this uncommon etiology of glomerular disease to put Hg on the clinician's radar for a reversible cause of nephrotic syndrome.

## Disclosure

All the authors declared no competing interests.

## Supplementary Material

Supplementary File (PDF)

**Supplementary References.**

## References

[1] Debiec H., Lefeu F., Kemper M.J. (2011). Early-childhood membranous nephropathy due to cationic bovine serum albumin [published correction appears in *N Engl J Med.* 2011;365:477] [published correction appears in *N Engl J Med.* 2014;370:886]. N Engl J Med.

[2] Spain R.I., Andeen N.K., Gibson P.C. (2021). Lipoic acid supplementation associated with neural epidermal growth factor-like 1 (NELL1)-associated membranous nephropathy. Kidney Int.

[3] Gao Z., Wu N., Du X. (2022). Toxic nephropathy secondary to chronic mercury poisoning: clinical characteristics and outcomes. Kidney Int Rep.

[4] Aymaz S., Gross O., Krakamp B., Ortmann M., Dienes H.P., Weber M. (2001). Membranous nephropathy from exposure to mercury in the fluorescent-tube-recycling industry. Nephrol Dial Transplant.

[5] Kumar M.N., Priyamvada P.S., Chellappan A. (2020). Membranous nephropathy associated with indigenous Indian medications containing heavy metals. Kidney Int Rep.

[6] Qin A.B., Yu X.J., Wang S.X., Zhou F.D., Zhao M.H. (2021). Unveiling the features of mercury-associated minimal change disease: comparison with primary minimal change disease. Kidney Dis (Basel).

[7] Li S.J., Zhang S.H., Chen H. (2010). Mercury-induced membranous nephropathy: clinical and pathological features. Clin J Am Soc Nephrol.

[8] Qin A.B., Su T., Wang S.X., Zhang F., Zhou F.D., Zhao M.H. (2019). Mercury-associated glomerulonephritis: a retrospective study of 35 cases in a single Chinese center. BMC Nephrol.

[9] Oliveira D.B., Foster G., Savill J., Syme P.D., Taylor A. (1987). Membranous nephropathy caused by mercury-containing skin lightening cream. Postgrad Med J.

