# Supplementary material for: What Can Mercury Teach Us About Membranous Nephropathy and Minimal Change Disease?
Source: Kidney Int Rep. 2022 Apr 22;7(6):1157–60. doi: 10.1016/j.ekir.2022.04.078 (PMC9174039; doi:10.1016/j.ekir.2022.04.078)
Supplement: Supplementary File (PDF) [file mmc1.pdf]

Supplemental References:

S1. Kibukamusoke JW, Davies DR, Hutt MS. Membranous nephropathy due to skin-lightening cream. Br Med J. 1974;2(5920):646-7.

S2. Soo YO, Chow KM, Lam CW, Lai FM, Szeto CC, Chan MH, et al. A whitened face woman with nephrotic syndrome. Am J Kidney Dis. 2003;41(1):250-3.

S3. Tang HL, Mak YF, Chu KH, Lee W, Fung SK, Chan TY, et al. Minimal change disease caused by exposure to mercury-containing skin lightening cream: a report of 4 cases. Clin Nephrol. 2013;79(4):326-9.

S4. Zhang L, Liu F, Peng Y, Sun L, Chen C. Nephrotic syndrome of minimal change disease following exposure to mercury-containing skin-lightening cream. Ann Saudi Med. 2014;34(3):257-61.

S5. Doshi M, Annigeri RA, Kowdle PC, Subba Rao B, Varman M. Membranous nephropathy due to chronic mercury poisoning from traditional Indian medicines: report of five cases. Clin Kidney J. 2019;12(2):239-44.

S6. Berglund A, Pohl L, Olsson S, Bergman M. Determination of the rate of release of intra-oral mercury vapor from amalgam. J Dent Res. 1988;67(9):1235-42.

S7. Wilson VK, Thomson ML, Holzel A. Mercury nephrosis in young children, with special reference to teething powders containing mercury. Br Med J. 1952;1(4754):358-60.

S8. Becker CG, Becker EL, Maher JF, Schreiner GE. Nephrotic syndrome after contact with mercury. A report of five cases, three after the use of ammoniated mercury ointment. Arch Intern Med. 1962;110:178-86.

S9. Burston J, Darmady EM, Stranack F. Nephrosis dur to mercurial diuretics. Br Med J. 1958;1(5082):1277-9.

S10. Yawei S, Jianhai L, Junxiu Z, Xiaobo P, Zewu Q. Epidemiology, clinical presentation, treatment, and follow-up of chronic mercury poisoning in China: a retrospective analysis. BMC Pharmacol Toxicol. 2021;22(1):25.

S11. Somers EC, Ganser MA, Warren JS, Basu N, Wang L, Zick SM, et al. Mercury Exposure and Antinuclear Antibodies among Females of Reproductive Age in the United States: NHANES. Environ Health Perspect. 2015;123(8):792-8.

S12. Zalups RK. Early aspects of the intrarenal distribution of mercury after the intravenous administration of mercuric chloride. Toxicology. 1993;79(3):215-28.

S13. Häggqvist B, Hultman P. Effects of deviating the Th2-response in murine mercury-induced autoimmunity towards a Th1-response. Clin Exp Immunol. 2003;134(2):202-9.

S14. Ochel M, Vohr HW, Pfeiffer C, Gleichmann E. IL-4 is required for the IgE and IgG1 increase and IgG1 autoantibody formation in mice treated with mercuric chloride. J Immunol. 1991;146(9):3006-11.

S15. Fuhr BJ, Rabenstein DL. Nuclear magnetic resonance studies of the solution chemistry of metal complexes. IX. The binding of cadmium, zinc, lead, and mercury by glutathione. J Am Chem Soc. 1973;95(21):6944-50.

S16. Pollard KM, Hultman P. Effects of mercury on the immune system. Met Ions Biol Syst. 1997;34:421-40.

S17. Aten J, Veninga A, Bruijn JA, Prins FA, de Heer E, Weening JJ. Antigenic specificities of glomerular-bound autoantibodies in membranous glomerulopathy induced by mercuric chloride. Clin Immunol Immunopathol. 1992;63(1):89-102.

S18. Abedi-Valugerdi M, Hu H, Möller G. Mercury-induced renal immune complex deposits in young (NZB x NZW)F1 mice: characterization of antibodies/autoantibodies. Clin Exp Immunol. 1997;110(1):86-91.

S19. Bigazzi PE. Metals and kidney autoimmunity. Environ Health Perspect. 1999;107 Suppl 5(Suppl 5):753-65.

S20. Schwenk M, Klein R, Templeton DM. Immunological effects of mercury (IUPAC Technical Report). Pure Appl Chem. 2009;81(1):153-67.
S21. Bernard A, Lauwerys R, Ouled Amor A. Loss of glomerular polyanion correlated with albuminuria in experimental cadmium nephropathy. Arch Toxicol. 1992;66(4):272-8.
S22. Lin L, Sun D, Wang W, Pan X, Ren H, Zhang W, et al. The relationship between membranous nephropathy and heavy metals. Nephrology Dialysis Transplantation. 2017;32:iii225.
S23. Hall CL, Jawad S, Harrison PR, MacKenzie JC, Bacon PA, Klouda PT, et al. Natural course of penicillamine nephropathy: a long term study of 33 patients. Br Med J (Clin Res Ed). 1988;296(6629):1083-6.
S24. Radford MG, Holley KE, Grande JP, Larson TS, Wagoner RD, Donadio JV, et al. Reversible membranous nephropathy associated with the use of nonsteroidal anti-inflammatory drugs. J Am Med Assoc. 1996;276(6):466-9.
S25. Yoshida A, Morozumi K, Suganuma T, Sugito K, Ikeda M, Oikawa T, et al. Clinicopathological findings of bucillamine-induced nephrotic syndrome in patients with rheumatoid arthritis. Am J Nephrol. 1991;11(4):284-8.
S26. Caza TN, Al-Rabadi LF, Beck LH, Jr. How Times Have Changed! A Cornucopia of Antigens for Membranous Nephropathy. Front Immunol. 2021;12:800242.
S27. Ye BJ, Kim BG, Jeon MJ, Kim SY, Kim HC, Jang TW, et al. Evaluation of mercury exposure level, clinical diagnosis and treatment for mercury intoxication. Ann Occup Environ Med. 2016;28:5.
